# Flood Frequency and Duration Drive the Aquatic-Terrestrial Pesticide Transfer to Riparian Root-Zone Soil: A Mesocosm Study

**DOI:** 10.1007/s00244-026-01190-9

**Published:** 2026-04-08

**Authors:** Franziska Fiolka, Alessandro Manfrin, Franziska Middendorf, Stephane Mutel, Collins Ogbeide, María José Gormaz-Aravena, Miyako Molly Briggs, Jakob Wolfram, Clara Mendoza-Lera, Ralf Schulz

**Affiliations:** iES Landau, Institute for Environmental Sciences, RPTU Kaiserslautern- Landau, Fortstrasse 7, 76829 Landau, Germany

## Abstract

The aquatic-terrestrial transition areas, riparian zones, recognized as biodiversity hotspots, are increasingly subjected to various stressors, including chemical contaminants such as pesticides. These compounds can be transported into the riparian zone, from surrounding agricultural fields, e.g., via surface runoff, but also from the aquatic environments, e.g. during flooding events. However, little is known about how flood frequency and duration affect this aquatic-terrestrial transfer. To investigate the flood-mediated pesticide transport, we conducted an experiment in 16 flow-through unit mesocosms, each unit containing a stream supplied with water from an agriculturally influenced river, and an adjacent riparian area. We simulated flooding events with varying duration of three, seven and fourteen days, repeated four times between May through September. We sampled river water every week and riparian root-zone soil 24 h after each flooding event and analysed organic pesticides using high-performance liquid chromatography coupled with tandem mass spectrometry via electrospray ionization. We found that six pesticides were detected exclusively in riparian root-zone soil following four repeated flooding events. Our findings indicate that both longer flood durations and repeated flooding events tend to increase the total concentration of pesticides in the riparian root-zone soil. These results demonstrate that flooding promotes the movement of pesticides from streams into adjacent riparian areas. As flood frequency and intensity are expected to increase due to climate change, the significance of this transport pathway is likely to increase, with potential consequences for riparian biodiversity and habitat quality.

**Supplementary Information:**

The online version contains supplementary material available at 10.1007/s00244-026-01190-9.

## Introduction

Riparian zones function as transitional areas where aquatic and terrestrial ecosystems constantly exchange energy and matter, including contaminants, such as pesticides (Junk et al. 1989; Schulz et al. 2015). Pesticides can be introduced into surface waters through various sources, including surface runoff or spray-drift from adjacent agricultural fields (Berenzen et al. 2005; Schulz 2001). Riparian areas as aquatic-terrestrial transition zones may, however, also function as buffer zones for pesticides between agricultural fields and adjacent water bodies (Aguiar et al. 2015; Rasmussen et al. 2011). The buffer capacity of riparian areas is strongly influenced by local geomorphic features such as erosion rills, which allow runoff to reach the surface water and thereby may reduce the buffering capacity of riparian areas for pesticides (Stehle et al. 2016). However, pesticides can also be transported back from the water bodies to the riparian area via abiotic and biotic pathways (Schulz and Bundschuh 2020). The biotic pathway via aquatic emerging insects has been shown to transport organic pesticides (Kraus et al. 2021; Roodt et al. 2023) and various other contaminants (Kraus et al. 2014). On the other hand, flooding can serve as an abiotic pathway of aquatic to terrestrial pesticide transport (Schulz et al. 2015).

Flood-mediated transfer of pesticides has been shown for basal ecosystem components, in riparian soil and plants, both following extreme flooding (Stachel et al. 2006) and after moderate natural flooding events (Fiolka et al. 2024). Previous studies found higher concentrations of Polybrominated Diphenyl Ether (PBDE) in soil with a history of flooding compared to soil from non-flood-prone sites (Lake et al. 2011), and for Ethofumesate in soils of low-lying floodplain sampling points of the River Elbe (Karlsson et al. 2020). However, for Simazine, opposite results were found, with highest concentrations measured in plateau soils compared to low-lying sampling points (Karlsson et al. 2020). This suggests that there are still considerable knowledge gaps with regard to the importance of flooding versus other processes for contaminant transport in larger rivers. Additionally, the plant-specific uptake and transfer of flood-mediated contaminants (Stiegler et al. 2022; Fiolka et al. 2024) needs to be taken into account, highlighting the need for taxa-specific sampling. In order to further explore the transfer of organic pesticides to riparian areas, we need to consider the flooding scenarios that occur in nature, including varying frequency and duration.

To assess the effect of flood frequency and duration on the organic pesticide transfer into riparian root-zone soil, we conducted a mesocosm experiment in which we simulated four flooding events from May through September 2023, one every 38 days. At each flooding event, we defined four flood durations 3, 7 and 14 days, including a control in which no flooding was applied (0 days of flooding). We hypothesize that (i) flooding events can transport pesticides from the stream into the riparian root-zone soil. We further hypothesize that (ii) there is a positive correlation between the flood-mediated pesticide transfer and the flood frequency and duration, with longer and more frequent flooding events leading to higher pesticide concentrations in riparian root-zone soil.

## Methods

### RSM Facility and Flooding Scheme

**Fig. 1:**
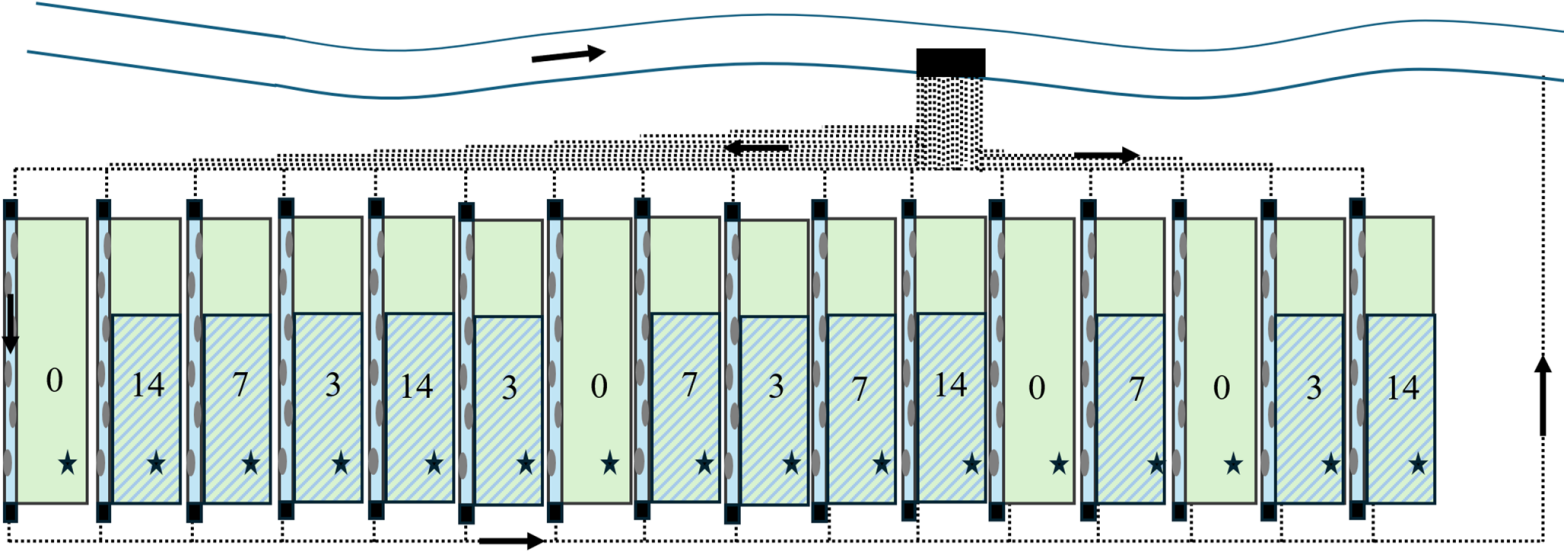
Riparian Stream Mesocosm (RSM) facility with its 16 independent units, each equipped with a riparian area (green) and a stream with six lateral gravel bars (grey areas). Flooded riparian areas are marked in blue, with the sampling spots marked with a star and flood durations indicated by a number (0-,3-,7-,14-days of flooding). Each unit is independently fed with water from the nearby River Queich through a pump-pipe system. Figure adapted from Rovelli et al. (2024)

The experiment was conducted at the Riparian Stream Mesocosm facility (RSM) in Landau, Germany (Rovelli et al. 2024) (Fig. 1). The facility consists of 16 spatially independent replicated aquatic-terrestrial mesocosm units each 15.15 ± 0.10 m long. Each unit consists of a 0.71 ± 0.06 m wide stream and a 3.70 ± 0.10 m wide riparian area situated at the left side of each stream (Fig. 1). Every unit is a flow-through system supplied with water from the nearby, agriculturally impacted, fourth order, River Queich (Schemmer et al. 2024). The River Queich originates in the Palatinate Forest biosphere reserve and flows through viticulture and urban areas upstream the RSM facility. River water is pumped for each mesocosm unit independently (BADU Eco Motion; SPECK Pumpen GmbH, Germany) and each riparian area can be individually flooded. The average water level in each stream was 8.6 ± 1.1 cm during normal flow conditions with 4.2 ± 0.9 L s^− 1^ of discharge and an average flow velocity of 0.2 ± 0.02 m s^− 1^ (Table 1).

Between May and September 2023, a total of four flooding events were experimentally simulated at the RSM, with one flooding event every 38 days. The experimental period from May to September covers the main pesticide application season in the surrounding agricultural areas. We defined four levels of duration that were repeated four times: (1) no flooding for 38 days (i.e., control), (2) three days of flooding and 35 days no flooding, (3) seven days of flooding and 31 days no flooding, and (4) 14 days of flooding and 24 days of no flooding. The treatments were assigned to the mesocosm units following a randomized block design (Fig. 1). The riparian areas had not experienced flooding at least 20 years prior to the start of the study. To implement the flooding, we increased the overall stream water level by raising the drainpipe at the stream outlet, subsequently flooding the riparian area, while still maintaining the stream flow. At the end of each corresponding flooding event, the height of the outlet drainpipe was lowered and the water level slowly returned to non-flooding levels and subsequent drainage of the riparian area. The flooding inundated 60% of the downstream area covering the entire width of the riparian area (370 ± 10 cm width; 910 ± 10 cm length), with 9 ± 2 cm maximum water depth (Fig. 1).

During flooding, we assessed in each unit preferential flow, discharge, and water residence time (Table 1) via discrete slug salt injections (see Rovelli et al. 2024). During flooding, mean flow velocity decreased from 0.2 ± 0.02 m s^− 1^ to 0.1 ± 0.01 m s^− 1^. The mean arrival time (MAT) of water increased ~ 2 times compared to the non-flooded conditions, indicating that there was exchange between the preferential flow in the stream section of the units and the flooded riparian area. Additionally, transient storage calculated as v/vMAT following Drummond et al. (2016) and Mendoza-Lera et al. (2019), indicated that transient storage increased during flooding by about 10%.

**Table 1:** Discharge, velocity, mean arrival time (MAT), MAT-integrated velocity (vMAT), and the ration v/v MAT during flooded and normal flow conditions (non-flooding) in the channel area

	Discharge (L s^− 1^)	Velocity (m s^− 1^)	MAT (m s^− 1^)	vMAT (m s^− 1^)	v/vMAT
Flood conditions	3.59 ± 1.59	0.10 ± 0.02	273.88 ± 52.88	0.06 ± 0.01	1.25 ± 0.15
Normal flow conditions	4.25 ± 0.88	0.21 ± 0.02	125.11 ± 12.79	0.12 ± 0.01	1.15 ± 0.03

### Sampling Scheme and Sample Preparation

We sampled riparian root-zone soil 24 h following the end of each respective flooding event. For each sampling, we uprooted a separate individual of common grass species (*Elymus* spp.) located at the sampling site in each flume (Fig. 1). The soil attached to the uprooted plant individual, an integrative sample of the soil between 0 and 20 cm depth, was shaken into a clean plastic bag and frozen at − 20 °C. Samples of the river water were taken to reflect the baseline pesticide load as grab water samples in 20 mL glass vials every week and frozen at − 20 °C for subsequent pesticide analysis.

Root-zone soil samples were transferred into falcon tubes and freeze dried at − 57 °C for a minimum of 20 h. Freeze-dried root-zone soil samples were then sieved using a 2 mm stainless steel sieve. Five grams of the root-zone soil sample were spiked with 50 µL Imidacloprid D4 (10 mg L^− 1^) as a procedural internal standard for quality control purposes and extracted using a previously published acetonitrile-based extraction method (Bakanov et al. 2023). A matrix-matched calibration curve with eight calibration points from 0.025 to 100 µg L^− 1^ was prepared using laboratory internal soil as blank material, in addition to matrix and solvent blanks. Frozen water samples were defrosted at 7 °C for 24 h and centrifuged at 15,200 rpm for 10 min (VWR Mega Star 1.6R centrifuge, Germany). A 350-µL aliquot of the supernatant was diluted with 150 µL of methanol containing an internal standard (Thiamethoxam-D3, 1.5 µg L^− 1^) and 0.3% formic acid (Roodt et al. 2023). A calibration series with eight concentrations covering the range from 0.0005 to 2 µg L^− 1^ was prepared using LC-grade water, in addition to solvent blanks.

### Pesticide Measurements

Pesticides were measured in water and root-zone soil samples using high performance liquid chromatography coupled to a triple quadrupole mass spectrometer by electrospray ionization (HPLC-ESI-MS/MS). Analytical standards were obtained from Restek (Bad Homburg, Germany) and solvents (LC–MS Grade) were purchased from Honeywell (Seelze, Germany). The HPLC elution gradient and mass spectrometry parameters were taken from Bakanov et al. (2023). Briefly, 1 µL root-zone soil sample volume was separated on a ZORBAX Eclipse Plus C18 (3.0 ID × 150 mm, 2.7 micron) column using a gradient elution of water and methanol containing 0.1% formic acid and 4 mM ammonium formate. At least two characteristic MRM-transitions were acquired for each analyte. Full details of the instrument parameters used for the measurements are provided in the SI (see Online Resource Table S1). The method used for the extraction and analyses of root-zone soil have been reported elsewhere and quantifies 96 insecticides, fungicides, and herbicides (Bakanov et al. 2023). Water samples were analysed for 70 pesticides using a direct injection method (Roodt et al. 2023). For the identification of analytes, the ratios of qualifier and quantifier peak areas had to match the calibration standard within ± 20%, and retention times were required to be within ± 0.05 min. The lowest concentration measured within the calibration curve that fulfilled all above stated criteria was accepted as limit of quantification (LOQ, see Online Resource Table S2). None of the targeted analytes were detected in any of the respective solvent blank samples.

### Root-Zone Soil Properties (pH, Conductivity, Water Content)

Root-zone soil pH, electrical conductivity and water content were measured in samples of three randomly selected mesocosm units for the lowest and highest flooding treatment, as well as for no flooding treatment (control, 3 and 14 days of flooding), after one and four flooding events. Samples were defrosted for five days at 6 °C. The pH measurements were conducted in milli-Q water and 0.01 M CaCl_2_ solution by adding 25 mL to 12 g wet root-zone soil sample. The pH reading was taken after 30 min (multiparameter analyser Consort C863). The specific conductivity was measured in the water-soil suspension (multiparameter analyser Consort C863). The root-zone soil samples were dried at 105 °C for five days and the dry weight was recorded to calculate the water content. The root-zone soil characteristics are reported in Online Resource Table S3.

### Data Analysis

A generalized linear mixed model (GLMM) using the glmmTMB function from the glmmTMB package (version 1.1.12, Brooks et al. 2017) with a Tweedie distribution (version 2.3.5, Dunn 2022) and log-link function was used to assess the effects of flood duration (days flooded), flood frequency (flood), and their interaction on the total pesticide concentration in root-zone soil samples (R version 4.5.1, R Core Team 2025). Random intercepts for unit identity were included to account for repeated measurements, although the estimated variance between units was negligible (variance = 2.85 × 10^− 9^). We conducted a post-hoc analysis using estimated marginal trends derived from the fitted GLMM. Estimated marginal trends for days flooded were computed separately for each level of flooding using the emtrends function from the emmeans package (version 1.11.2, Lenth 2025). Model diagnostics were performed using the DHARMa package (version 0.4.7, Hartig 2024). Residuals showed no significant deviations from uniformity (*p* = 0.762), dispersion (*p* = 0.584), or outliers (*p* = 1.0). In the residuals-versus-predicted plot, a slight deviation was detected in the upper quantiles (DHARMa adjusted quantile test: *p*-value < 0.05), suggesting a minor mismatch in model fit at higher predicted values. However, no funnel-shaped or systematic patterns were observed, and the deviation was considered negligible. Multicollinearity was ruled out based on low variance inflation factors (VIF < 1.1) from a Gamma GLM fitted to non-zero responses. The random intercept for unit was retained to account for repeated measures, despite a slightly lower AIC in the fixed-effects-only model.

## Results and Discussion

### Flood-Mediated Transfer of Pesticides into Riparian Root-Zone Soil After Frequent Flooding Events

We detected six pesticides exclusively in riparian root-zone soil after four flooding events (Table 2). All pesticides detected in riparian root-zone soil were present in the flood water, measured in the River Queich, during flooding events (Table 2). The presence of fungicides and insecticides in the river water is likely due to the agricultural land-use in the River Queich’s catchment (Schemmer et al. 2024). The total pesticide concentrations ranged from 0.003 to 0.292 µg L^− 1^ suggesting a high weekly fluctuation. The water concentrations of the 31 detected pesticides reported in the present study align with the median pesticide concentration in the River Queich of 0.006 µg L^− 1^ measured in 2021 (Ogbeide et al. 2025). 19% of the pesticides quantified in river water were also present in riparian root-zone soil after repeated flooding events at the end of the season in September (see Table S2). Twenty-five pesticides were present in flood water at least once in the weekly grab samples between May and September and were not quantified in riparian root-zone soil (see Table S2). It is worth noting here that our design generated flooding independent of precipitation patterns (Merz and Blöschl 2003) so that we could detect the actual effect of river flooding, and that under natural flooding conditions concentrations and presence of contaminants might be elevated (Halbach et al. 2021). Additionally, the concentrations reported in the present study represent inputs from a controlled number of flooding events in a mesocosm facility, whereas in natural floodplains, riparian areas may be contaminated via multiple exposure pathways, including surface water runoff from adjacent agricultural fields (Schulz 2001). Among the pesticides detected in river water, Fluopyram was detected in 92% of the water samples, followed by Isoproturon (69%), S-metolachlor (65%), Metalaxyl (62%), Metrafenone (54%) and Acetamiprid (54%). Fluopyram, the only pesticide detected in both flooded and non-flooded units, displayed higher average and maximum concentrations in flooded riparian root-zone soils compared to control riparian root-zone soil after repeated flooding events and also from the initial sampling prior to flooding (Table 2). The fungicide Fluopyram detected in the non-flooded units likely represent background contamination through exposure other than flooding, in fact Fluopyram is one of the most frequently detected pesticides in soil samples from a landscape-wide contamination study in the areas surrounding the mesocosm facility (Mauser et al. 2025).

Among the six pesticides being present exclusively in flooded riparian root-zone soil, we detected four fungicides (F), one herbicide (H), and one insecticide (I) (Table 2). The number of pesticides detected in root zone soils in the present study is about half that reported in a Europewide soil monitoring study with over 3,300 sites, around 70% of which were cropland, targeting 118 pesticide residues and reporting thirteen pesticides detected in more than 10% of sites with a limit of quantification of 0.001 mg kg^− 1^ (Franco et al. 2024). On the other hand, the number of pesticides detected in this study is about twice the number reported in another Europe-wide study on pesticide residues in agricultural topsoil, which targeted 76 pesticides in 317 sites and detected a median of three pesticides, with seven pesticides occurring in more than 10% of soil samples (Silva et al. 2019). The number of pesticides detected in this study is therefore within the same order of magnitude as the median pesticide numbers reported for agricultural soil, although pesticide concentrations in riparian root-zone soils are at least three orders of magnitude lower compared to agricultural soils (Silva et al. 2019). Flooding events therefore appear to lead to an assimilation of the pesticide profile in riparian root-zone soils comparable to agricultural soil, even though the presented pesticide concentrations reported in riparian areas are considerably lower compared to crop soils. Therefore, the ecological implications of pesticide mixtures in riparian root-zone soil will be different from those in agricultural landscapes because of differences in exposure pathways and concentrations. In fact, the measured concentrations of the individual pesticides in riparian root-zone soil are more than five orders of magnitude below the chronic NOEC (reproduction endpoint) for earthworms, which constitute an important component of soil fauna (Edwards 2004). By contrast, in a study with similar number of pesticides but substantially higher concentrations, risk indicator calculations for soils from the European LUCAS soil monitoring exceeded the critical value of one at 14% of the monitoring sites (Franco et al. 2024). A meta-analysis found that soil fauna communities were negatively affected by pesticides in terms of abundance and diversity (Beaumelle et al. 2023), as well as altered microbial functions (Köninger et al. 2026). It was found that both, pesticide mixtures exert stronger negative effects on soil communities than exposure to single substances (Beaumelle et al. 2023), and that risk indicator calculations in soils were mainly driven by a single substance (Franco et al. 2024). Therefore, the ability of flooding events to transport pesticide mixtures at low concentrations into riparian root-zone soils as shown in the present study, might be relevant for soil fauna communities in frequently flooded and agriculturally impacted areas, as our results suggest that the implications for the communities will vary as a function of flooding.

We detected six of the 31 pesticides found in flood water in riparian root-zone soil after repeated flooding events at the end of the season in September (see Table S2). While the present study targets pesticide parent compounds and focuses on the short-term changes after flooding, a field study in the Upper Elbe River found a flood-mediated transfer of transformation products without being able to quantify the respective parent compounds atrazine and terbuthylazine in the floodplain (Karlsson et al. 2020). Therefore, both parent compounds and transformation products might be transported flood-mediated or present in flooded soils, depending on their physicochemical properties and environmental conditions. The six pesticides reported as potentially flood-mediated in the present study display low (≤ 10 mg L^− 1^; Azoxystrobin, Metrafenone, Boscalid), moderate (< 1000 mg L^− 1^; Spiroxamine, Isoproturon) and high solubility in water (Acetamiprid) (PPDB 2025). This variability in solubility suggests that water solubility alone may not be a reliable indicator of a pesticide’s susceptibility to flood-mediated transfer. This contrasts with a study conducted around the River Elbe, which detected two pesticides and suggested a flood-mediated transfer of Ethofumesate, a moderately water-soluble pesticide, but not of Simazine, a pesticide with low water solubility (Karlsson et al. 2020). Other physicochemical properties of the compounds that might influence the flood-mediated transfer of the respective pesticide could be the organic carbon adsorption coefficient (K_oc_), vapor pressure, and environmental half-life (Payraudeau et al. 2025). However, the flood-mediated pesticides identified in this study span a wide range of values for these properties, from non-mobile to moderately mobile (K_oc_ > 75 mL g^− 1^), low to moderately volatile (< 10 mPa), slow to fast water-sediment degradation (< 365 days), and the full range from fast to stable water phase only degradation (PPDB 2025). Thus, none of the reported individual physicochemical parameters appear to consistently predict flood-mediated transport for the measured pesticides in the present study. Similarly, the presence of pesticides detected in soil and vegetation in remote alpine environments (Brühl et al. 2024) nor in passive air samples (Zaller et al. 2022) could not be linked to their physicochemical parameters such as vapour pressure. Overall, the flood-mediated pesticide transfer likely depends on a combination of factors, including chemical binding potential (sorption), solubility, mobility, and biotic degradation processes, as described for pesticide fate in the soil-sediment water phase (Vryzas 2018). Particularly under anaerobic conditions, which often occur in saturated soils following flooding, degradation dynamics are altered. This has been shown for the herbicide Imazapyr, degrading up to three times faster under anaerobic conditions (Wang et al. 2006), as well as for the insecticide neonicotinoid Acetamiprid, with a ten times greater half-life during dry conditions (Gupta and Gajbhiye 2007). Similarly to the airborne transport of pesticides, this highlights a critical knowledge gap in understanding and predicting which pesticides are most prone to flood-mediated transport based on currently used parameters.

**Table 2:** Concentration of pesticides (F = fungicide, I = insecticide, H = herbicide) measured in water and in riparian root-zone soil after four flooding events in flooded and non-flooded areas indicated by mean ± STD and maximum values

Compound (compound class)	River water		Riparian root-zone soil			
	Concentration (µg L^− 1^)		Concentration (µg kg^− 1^) Flooded		Concentration (µg kg^− 1^) Control (0-day flood)	
	Mean ± STD	Max	Mean ± STD	Max	Mean ± STD	Max
Spiroxamine (F)	0.001 ± 0.0006	0.002	0.02 ± 0.05	0.17	0	0
Isoproturon (H)	0.005 ± 0.004	0.019	0.01 ± 0.02	0.06	0	0
Boscalid (F)	0.03 ± 0.02	0.05	0.10 ± 0.18	0.53	0	0
Azoxystrobin (F)	< LOQ	< LOQ	0.04 ± 0.04	0.09	0	0
Acetamiprid (I)	0.003 ± 0.002	0.007	0.01 ± 0.02	0.08	0	0
Metrafenone (F)	0.008 ± 0.02	0.06	0.06 ± 0.1	0.32	0	0
Fluopyram (F)	0.008 ± 0.02	0.11	0.14 ± 0.14	0.4	0.1 ± 0.1	0.22

## Influence of Flood Frequency and Duration on Flood-Mediated Pesticide Transfer

**Fig. 2:**
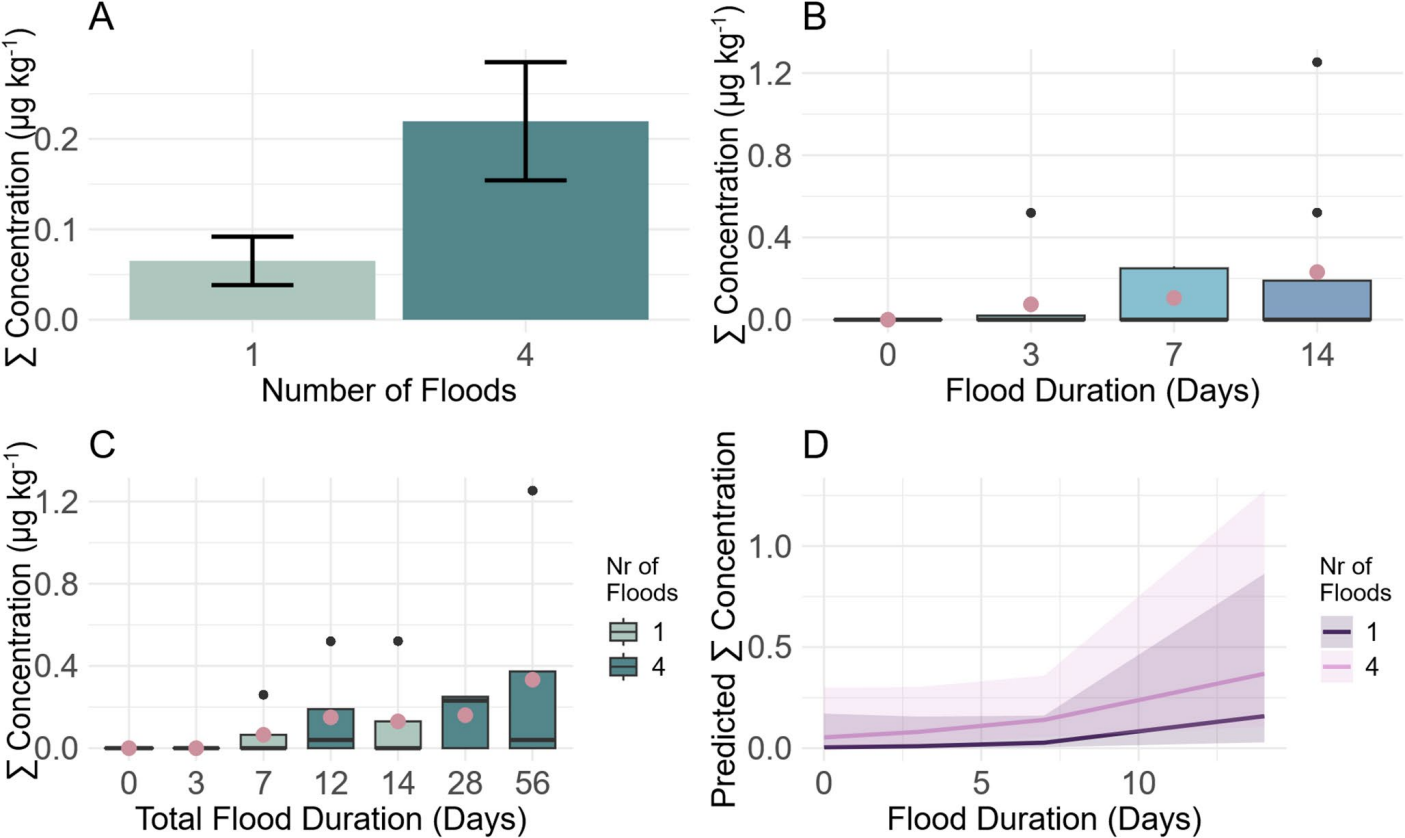
Total pesticide concentration (µg kg^− 1^) of the six flood-mediated pesticides in riparian root-zone soil displayed for (**A**) one or four repeated flooding events as Mean ± CI, (**B**) 0, 3, 7 and 14 days of flood duration, boxplot displaying median (black line) with mean (pink dot), (**C**) total flood duration (days flooded multiplied by times flooded) as median (black line) and mean (pink dot) for soil samples measured after one flood (light) and after four floods (dark), (**D**) days of flood duration during one flood (dark) and during four flood (light) as predicted values ± CI (line± area)

The total pesticide concentration of the six flood-mediated pesticides in riparian root-zone soil after four flooding events tended to increase over three-fold compared to after one flooding event (Fig. 2A, glmmTMB summary: Estimate = 2.44, SE = 2.04, z = 1.20, *p* = 0.23). For each specific flood duration (3, 7, 14 days), the measured total pesticide concentration is higher after four flooding events, compared to after one flooding event (Fig. 2C, light vs. dark bars). This reported three-fold increase is in accordance with previous field studies. Fiolka et al. (2024) measured a ten-fold higher pesticide concentration in regularly flooded riparian soil of small and medium sized streams in Germany compared to rarely flooded soil. Lake et al. (2011) measured in farms along the River Trent in the UK almost triple the median PBDE concentrations in soil repeatedly exposed to flooding compared to soil not flooded. On the other hand, a 4-year survey following a catastrophic flood of the Elbe River in 2002, detected no elevated PCBs concentration in flooded soils compared to non-flooded soils, but for PAHs and DDT (Pulkrabová et al. 2008). Therefore, regular, repeated flooding events, lead to an increase in pesticide concentrations as shown in the present study in riparian root-zone soil of small sized streams. It was also shown that pesticide concentrations in flooded soils might decrease within days after individual flooding events but increase cumulatively with repeated flooding (Fiolka et al. 2025). This is especially relevant in the context of climate change, as flooding events in general are predicted to becoming more frequent in certain areas (Blöschl et al. 2019). Studies on riverine flood trends show that there is an increase in flood risk with modelled climate change scenarios of up to 220% within this century in river basins with upstream areas larger than 500 km^2^ (Alfieri et al. 2015). Especially summer floods seem to be particularly affected as precipitation events in summer are projected to increase in magnitude and frequency in the near future due to climate change (IPCC 2007).

Total pesticide concentrations of the six flood-mediated pesticides tend to increase with flood duration (Fig. 2B, glmmTMB summary: Estimate = 0.25, SE = 0.16, z = 1.59, *p* = 0.11). During the first flooding event, each day of additional flooding increases the total pesticide concentration in riparian root-zone soil by factor 1.29. For the fourth flooding event, each day of additional flooding increases the total pesticide concentration by factor 1.14. The increase in total pesticide concentration in riparian root-zone soil with additional days of flooding is therefore lower after four flooding events, however this effect does not reach significance (Fig. 2C, interaction). The duration of flooding events has received little attention in previous field or mesocosm studies on contaminants in flooded areas, with a main focus on the recurrence of flooding events (Stachel et al. 2006; Pulkrabová et al. 2008, Fiolka et al. 2024). However, the present study could show an increase of pesticide concentration with every additional day of flooding (Fig. 2D). The higher pesticide concentrations due to longer flooding periods could be related to the increased contact time of the moving flood water with the soil during longer flooding periods. This is also supported by the increase in mean arrival time of water in the present study, which show an exchange of the water present in the flooded terrestrial areas during the time of flooding (Table 1). This results in longer exposure of the soil to the pesticides via flood water, which may explain the higher pesticide concentrations in the soil. However, also flooding events without dynamic water exchange in the floodplain could facilitate higher pesticide concentrations as function of the pesticide load in the water. This could be due to a higher sedimentation rate with longer standing water, where sediment deposits could transport pesticides, such as the glyphosate metabolite AMPA (Didoné et al. 2021). In addition, a decrease in leaching potential with increasing soil residence time, as shown for Imidacloprid, may also contribute (Oi 1999). The increase in flood-mediated pesticide transfer into riparian soil with longer flooding durations as shown in the present study might gain further relevance due to the proposed increased intensity of riverine flooding events due to climate change (IPCC 2023).

## Conclusion

Flooding represents an increasingly recognized yet understudied pathway for pesticide entry into terrestrial ecosystems, complementing known exposure pathways such as the biotic transfer via e.g. aquatic emerging insects. Although flood-mediated pesticide concentrations haven been shown to be below ecotoxicologically relevant concentrations, the increasing frequency and spatial extent of flooding events might lead to accumulation and chronic exposure in riparian areas. Especially in small to medium sized streams, the recurrent nature of flooding might be able to promote the transfer of pesticides to soils. Flooding events might be able to expose wide stretches of riparian areas with low-concentration pesticide mixtures, which might lead to chronic exposure of non-target organism in riparian areas. Moreover, riparian soil might act as a long-term sink for persistent pesticides, which might pose risk to essential soil functions and further might act as a source of contamination for the wider riparian area. Therefore, flooding as an exposure pathway should be considered as a potentially ecologically relevant pathway.

## Supplementary Information

Below is the link to the electronic supplementary material.

Supplementary Material 1

## Data Availability

The datasets generated during the current study are available from the corresponding author on reasonable request.
