# Supplementary material for: Flood Frequency and Duration Drive the Aquatic-Terrestrial Pesticide Transfer to Riparian Root-Zone Soil: A Mesocosm Study
Source: Arch Environ Contam Toxicol. 2026 Apr 8;90(3):21. doi: 10.1007/s00244-026-01190-9 (PMC13061809; doi:10.1007/s00244-026-01190-9)
Supplement: Supplementary file 1 — Supplementary Material 1 [file 244_2026_1190_MOESM1_ESM.docx]

**Supplementary Material**

**For**

**Flood Frequency and Duration Drive the Aquatic-Terrestrial Pesticide Transfer to Riparian Root-zone Soil: A Mesocosm Study**

Franziska Fiolka^1^, Alessandro Manfrin^1^, Franziska Middendorf^1^, Stephane Mutel^1^, Collins Ogbeide^1^, Maria Jose Gormaz Aravena^1^, Miyako Briggs^1^, Jakob Wolfram^1^, Clara Mendoza-Lera^1^, Ralf Schulz^1^

^1^ iES Landau, Institute for Environmental Sciences, RPTU Kaiserslautern-Landau, Fortstrasse 7, D-76829, Landau, Germany

This PDF File includes:

Table S1-S3

**Table S1:** Instrument specifications.

| Procedures of analytical analysis: Agilent 1260 Infinity II HPLC conditions. | |
| --- | --- |
| Parameter | Value |
| Column | Agilent ZORBAX Eclipse Plus C18. 3x150 mm. 2.7 µ |
| Column temperature | 45 °C |
| Injection volume (soil samples) | 1 µL |
| Injection volume (water samples) | 100 µL |
| Speed | Draw. 200 µl min^-1^ Eject: 400 µL min^-1^ |
| Needle wash | 12 seconds |
| Mobile phase | A) 98 % Water with 4 mM ammonium formate + 0.1 % acetic acid + 2% methanol  B) 98 % methanol with 4 mM ammonium formate + 0.1 % acetic acid + 2% water |
| Flow rate | 0.45 mL min^-1^ |
| Gradient program | Time B%  0 2  1.0 50  4.0 65  14.0 100  20.0 100  20.1 2 |
| Stop time | 20.1 minutes |
| Post time | 3 minutes |
| Procedures of analytical analysis: Agilent 6495C MS parameters for positive and negative ion method. | |
| Parameter | Value |
| Mass Spectrometer | Agilent 6495C with electrospray ionization (ESI) operated in multiple reaction monitoring mode |
| Ionization mode | Positive / Negative |
| Gas temperature | 250 °C; 11 L min^-1^ |
| Nebulizer | 38 psi |
| Sheath gas | 350 °C; 12 L min^-1^ |
| Capillary voltage | 3.000 V / 3000 V |
| Delta EMV | 200 |
| High-pressure iFunnel RF | 150 V / 150 V |
| Low-pressure iFunnel RF | 60 V / 60 V |
| MS1 and MS2 resolution | Unit |

**Table S2:** Limit of quantification (LOQ) for water samples and soil (values for targeted compounds), and list of compounds detected in water samples.

| Compound | LOQ (µg L^-1^) Water | Detected at least once May to September | LOQ (µg kg ^-1^) Soil |
| --- | --- | --- | --- |
| 2,4-D |  |  | 20 |
| Acetamiprid | 0.0005 | >LOQ | 0.1 |
| Aminopyralid | 0.01 | >LOQ | 2 |
| Azoxystrobin | 0.005 | <LOQ | 0.05 |
| Benalaxyl | 0.0005 | >LOQ | 0.05 |
| Bentazone | 0.001 | >LOQ | 0.05 |
| Bixafen | 0.001 |  | 0.1 |
| Boscalid | 0.005 | >LOQ | 0.2 |
| Bromoxynil |  |  | 2 |
| Carfentrazone-ethyl | |  | 2 |
| Chlorantraniliprole | 0.001 |  | 1 |
| Chloridazon | 0.01 | >LOQ | 0.05 |
| Chlortoluron | 0.0005 | >LOQ | 0.1 |
| Clomazone | 0.005 | <LOQ | 0.05 |
| Clothianidin |  |  | 0.05 |
| Cyantraniliprole | 0.001 | >LOQ | 2 |
| Cyazofamid | 0.005 | <LOQ | 1 |
| Cyflufenamid | 0.001 | >LOQ | 0.05 |
| Cymoxanil |  |  | 0.05 |
| Difenconazole | 0.005 | >LOQ | 20 |
| Diflufenican | 0.001 |  | 0.05 |
| Dimethenamid-P | 0.001 | >LOQ | 0.05 |
| Dimethoate | 0.001 | <LOQ | 0.05 |
| Dimoxystrobin | 0.0005 |  | 0.05 |
| Epoxiconazole | 0.005 |  | 1 |
| Ethofumesate | 0.01 |  | 1 |
| Etofenprox |  |  | 0.1 |
| Fenoxycarb | 0.005 |  | 0.05 |
| Fenpropimorph | |  | 0.05 |
| Fenpyroximate | |  | 0.05 |
| Fipronil | 0.0005 | >LOQ | 1 |
| Fipronil desulfinyl | 0.0005 |  | 1 |
| Fipronil sulfone | 0.001 |  | 1 |
| Flazasulfuron | 0.0005 |  | 1 |
| Flonicamid |  |  | 1 |
| Florasulam |  |  | 20 |
| Fluazinam |  |  | 1 |
| Fludioxonil | 0.005 |  | 1 |
| Flufenacet | 0.001 | <LOQ | 0.05 |
| Fluopicolid | 0.005 | >LOQ | 0.05 |
| Fluopyram | 0.0005 | >LOQ | 0.05 |
| Flupyradifurone | 0.001 |  | 0.1 |
| Fluroxypyr | 0.5 |  | 20 |
| Flurtamone | 0.0005 | <LOQ | 0.05 |
| Foramsulfuron | 0.05 |  | 2 |
| Hexythiazox |  |  | 0.05 |
| Imidacloprid | 0.05 | <LOQ | 0.2 |
| Indoxacarb |  |  | 1 |
| Iprovalicarb | 0.001 | >LOQ | 0.05 |
| Isoproturon | 0.001 | >LOQ | 0.05 |
| Kresoxim methyl | 0.0005 | >LOQ | 0.05 |
| Lenacil |  |  | 1 |
| Mandipropamid | 0.0005 | >LOQ | 0.05 |
| MCPA | 0.05 |  | 20 |
| Metalaxyl | 0.001 | >LOQ | 0.1 |
| Metamitron | 0.005 | >LOQ | 1 |
| Metazachlor | 0.0005 | <LOQ | 0.05 |
| Methiocarb | 0.0005 |  | 0.05 |
| Methiocarb-sulfoxide | 0.0005 |  | 0.05 |
| Methoxyfenozide | 0.05 |  | 0.05 |
| Metobromuron | 0.001 | >LOQ | 0.1 |
| Metolachlor-S | 0.0005 | >LOQ | 0.05 |
| Metrafenone | 0.001 | >LOQ | 1 |
| Myclobutanil | 0.001 | >LOQ | 1 |
| Napropamide | 0.0005 |  | 0.05 |
| Omethoate |  |  | 0.05 |
| Paclobutrazol | 0.0005 | >LOQ | 1 |
| Penconazole | 0.0005 | >LOQ | 0.05 |
| Pencycuron |  |  | 0.05 |
| Pendimethalin | |  | 1 |
| Picloram |  |  | 20 |
| Picoxystrobin |  |  | 0.05 |
| Pirimicarb | 0.0005 | >LOQ | 0.05 |
| Prochloraz |  |  | 1 |
| Propamocarb | 0.0005 | >LOQ | 0.05 |
| Propaquizafop | |  | 1 |
| Propyzamide | 0.0005 | <LOQ | 0.05 |
| Proquinazid | 0.0005 | >LOQ | 0.05 |
| Prosulfocarb | 0.001 | <LOQ | 0.1 |
| Prothioconazole-desthio | 0.005 | <LOQ | 20 |
| Pymetrozine |  |  | 1 |
| Pyraclostrobin | 0.0005 | <LOQ | 0.05 |
| Pyrimethanil | 0.05 |  | 1 |
| Quinmerac | 0.001 | <LOQ | 1 |
| Quizalofop |  |  | 20 |
| Spinosad A | 0.005 |  | 0.038 |
| Spinosad D |  |  | 0.48 |
| Spirotetramat | 0.001 |  | 0.05 |
| Spiroxamine | 0.0005 | >LOQ | 0.05 |
| Sulfoxaflor |  |  | 0.05 |
| Tebufenozide | 0.005 | <LOQ | 0.2 |
| Terbuthylazine | 0.0005 | >LOQ | 0.05 |
| Thiacloprid | 0.0005 | <LOQ | 0.05 |
| Thiamethoxam | 0.0005 | >LOQ | 0.05 |
| Trifloxystrobin | 0.0005 |  | 0.05 |
| Tritosulfuron |  |  | 0.05 |

**Table S3**: Riparian root-zone soil pH. conductivity and water content.

| Flooding event | Flood duration | pH H_2_O | Electrical conductivity (µS/cm) | pH CaCl_2_ | Water content (%) |
| --- | --- | --- | --- | --- | --- |
| 1 | 0 | 6.3 | 37.1 | 5.8 | 6.3 |
| 1 | 3 | 6.2 | 36.3 | 5.4 | 20.2 |
| 1 | 14 | 6.3 | 52.9 | 5.5 | 20.6 |
| 4 | 0 | 6.7 | 66.8 | 5.8 | 15.1 |
| 4 | 3 | 6.8 | 56.1 | 6.0 | 30.2 |
| 4 | 14 | 6.9 | 35.6 | 5.9 | 31.9 |
